# Bilayer MoSe_2_/HfS_2_ Nanocomposite as a Potential Visible-Light-Driven Z-Scheme Photocatalyst

**DOI:** 10.3390/nano9121706

**Published:** 2019-11-28

**Authors:** Biao Wang, Xiaotian Wang, Peng Wang, Tie Yang, Hongkuan Yuan, Guangzhao Wang, Hong Chen

**Affiliations:** 1 School of Physical Science and Technology, Southwest University, Chongqing 400715, China; wangxt45@126.com (X.W.); wangpeng9@swu.edu.cn (P.W.); yangtie@swu.edu.cn (T.Y.); yhk10@swu.edu.cn (H.Y.); 2 School of Electronic Information Engineering, Key Laboratory of Extraordinary Bond Engineering and Advanced Materials Technology of Chongqing, Yangtze Normal University, Chongqing 408100, China; wangyan6930@yznu.edu.cn; 3 Key Laboratory of Luminescent and Real-Time Analytical Chemistry, Ministry of Education, College of Chemistry and Chemical Engineering, Southwest University, Chongqing 400715, China

**Keywords:** MoSe_2_/HfS_2_, direct Z-scheme, photocatalytic water splitting, hybrid functional study

## Abstract

Visible-light-driven photocatalytic overall water splitting is deemed to be an ideal way to generate clean and renewable energy. The direct Z-scheme photocatalytic systems, which can realize the effective separation of photoinduced carriers and possess outstanding redox ability, have attracted a huge amount of interest. In this work, we have studied the photocatalytic performance of the bilayer MoSe_2_/HfS_2_ van der Waals (vdW) heterojunction following the direct Z-scheme mechanism by employing the hybrid density functional theory. Our calculated results show that the HfS_2_ and MoSe_2_ single layers in this heterojunction are used for the oxygen evolution reaction (OER) and hydrogen evolution reaction (HER), respectively. The charge transfer between the two layers brought about an internal electric field pointing from the MoSe_2_ layer to the HfS_2_ slab, which can accelerate the separation of the photoinduced electron–hole pairs and support the Z-scheme electron migration near the interface. Excitingly, the optical absorption intensity of the MoSe_2_/HfS_2_ heterojunction is enhanced in the visible and infrared region. As a result, these results reveal that the MoSe_2_/HfS_2_ heterojunction is a promising direct Z-scheme photocatalyst for photocatalytic overall water splitting.

## 1. Introduction

In order to set up a sustainable society, photocatalytic water splitting for hydrogen production has been deemed as an effective route to solve the problems of environmental pollution and energy shortage [1]. Owing to the groundbreaking work by Honda and Fujishima in 1972 [2], a variety of semiconductor materials have been extensively investigated to explore high-performance photocatalysts for water decomposition [3,4,5,6,7]. However, a majority of one-component photocatalysts, such as TiO_2_ and ZnO, can only utilize a small amount of the solar energy and the lifetimes of photoinduced electron–hole pairs in these materials are short, which leads to the problem that the photocatalytic efficiency is low and hampers their future applications [8,9]. For the sake of overcoming these disadvantages, many researchers have found that the construction of heterostructures, which are composed of different materials and can supply much greater control of the electronic and optical properties, is able to effectively enhance the catalytic activity [10,11,12,13,14,15].

In particular, great expectations have been placed on the Z-scheme photocatalytic mechanism—which includes two-step excitation and is illuminated by natural photosynthesis in plants [16]—for improving the utilization efficiency of sunlight [17,18,19,20,21,22,23,24]. Generally speaking, the Z-scheme photocatalytic system is made up of three parts: Catalysts for the hydrogen evolution reaction (HER), oxygen evolution reaction (OER), and the redox mediator for carrier migration [25]. According to this mechanism, although the isolated components cannot accomplish overall water splitting, the combined systems can decompose water into hydrogen and oxygen, which can broaden the scope of the promising photocatalysts. Moreover, the Z-scheme photocatalytic composites possess strong redox abilities and can benefit the separation of photogenerated carriers because of the occurrence of HER and OER on different layers [26]. Nevertheless, the redox mediators in Z-scheme photocatalysts may induce undesired back reactions and seriously affect the photocatalytic performance [27]. Z-scheme systems without mediators, which are named as direct Z-scheme photocatalysts, can avoid the problems caused by mediators and are easier to experimentally fabricate owing to their simpler structures. Therefore, the direct Z-scheme systems have been extensively studied [28,29,30,31,32].

Recently, various two-dimensional (2D) materials, such as graphene [33], g-C_3_N_4_ [34], MoSe_2_ [35], and HfS_2_ [36] have been predicated and fabricated [37]. Owing to the maximized specific surface area, high charge migration, and unique electronic properties derived from the quantum confinement effect, 2D materials and related heterostructures have attracted much attention as high-performance photocatalysts for water [38,39,40]. However, being limited by the band edge positions, the isolated MoSe_2_ and HfS_2_ monolayers can only be used for the HER and OER [41,42], respectively. Fortunately, the band edge positions of MoSe_2_ and HfS_2_ single layers present staggered-type (type II) band alignment characteristics [43,44], which are beneficial for establishing the Z-scheme composites. Therefore, we have designed MoSe_2_/HfS_2_ bilayer nanocomposites and studied their geometric structures, band structures, density of states, charge transfer, stress effect, and optical properties by employing the hybrid density functional theory.

## 2. Computational Method

In this work, all of the density functional theory calculations were employed with the Vienna ab initio simulation package (VASP) [45]. The frozen-core projector augmented wave (PAW) was used to describe the interaction between the core and valence electrons [46,47]. The generalized gradient approximation (GGA) [48] of the Perdew–Burke–Ernzerhof (PBE) exchange correlation functional [49] was adopted. The charge redistribution in the MoSe_2_/HfS_2_ heterojunction was considered by calculating the dipole correction [50]. For the sake of avoiding the undervaluation of bandgaps calculated by the PBE method, the electronic and optical properties of these referred materials were calculated by the Heyd–Scuseria–Ernzerhof (HSE) hybrid functional by adding a part of the exact exchange interaction [51]. An exact exchange contribution of 0.25 was used in this study. The long-range van der Waals (vdW) interaction of heterojunctions was described by the Grimmes DFT-D3 method [52]. A Γ-centered 7 × 7 × 1 K-point was used to sample the 2D Brillouin Zone [53]. The cutoff energy was set as 500 eV. The convergence criteria were less than 10^−5^ eV for total energy and 0.01 eV/Å for Hellman–Feynman force on each atom, respectively. A vacuum space of 20 Å was inserted perpendicular to the layers to separate the neighboring slabs of heterojunctions. The band edge positions of 2D materials were calculated by subtracting vacuum levels, which were obtained by averaging the values in the LOCPOT file; they were then applied for measuring the absolute positions of energy bands.

## 3. Results and Discussion

### 3.1. Structural Stability

**Figure 1:**
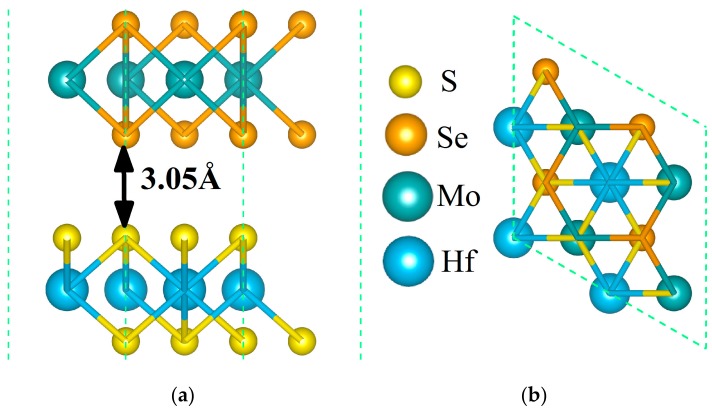
The equilibrium structure of the MoSe_2_/HfS_2_ nanocomposite. Orange and peacock-blue balls signify Se and Mo atoms in the MoSe_2_ monolayer; yellow and dodger-blue balls symbolize S and Hf atoms in the HfS_2_ single layer, respectively. The side view (**a**) and top view (**b**) of this heterojunction.

In this study, we chose the hexagonal MoSe_2_ and HfS_2_ monolayers, which are able to fit each other well. After the structural optimization, the lattice constants of MoSe_2_ and HfS_2_ single layers were 3.30 Å and 3.61 Å, respectively, which agree with the previous values [54]. Due to the small lattice mismatch of these single layers, we designed a heterojunction composed of 2 × 2 MoSe_2_ and HfS_2_ supercells. In order to probe into the steadiest stacked pattern, we studied various configurations. The steadiest configuration of the MoSe_2_/HfS_2_ heterojunction, whose equilibrium structure is presented in Figure 1, will be studied in the subsequent parts.

In addition, the interlayer spacing of the MoSe_2_/HfS_2_ heterobilayer was 3.05 Å, as displayed in Figure 1, which refers to the vertical distance from the nearest S atoms of the HfS_2_ layer to the Se atoms of the MoSe_2_ layer. This vertical separation between two layers is a typical vdW equilibrium distance, which is in accord with other 2D vdW heterostructures [11,44]. Thus, it was necessary to add the vdW corrections into our computation. The binding energy (E_b_) of the MoSe_2_/HfS_2_ heterojunction can be defined as this equation: E_b_ = E_MoSe2/HfS2_ − E _MoSe2_ − E _HfS2_, where E_MoSe2/HfS2_, E _MoSe2_, and E _HfS2_ stand for the total energy of this heterostructure, isolated MoSe_2_, and HfS_2_ monolayers, respectively. After calculations, the bound energy of the steadiest configuration of the MoSe_2_/HfS_2_ heterojunction is –0.27 eV, which means that this composite is stable.

### 3.2. Electronic Properties

The band structures of related materials were calculated by employing the hybrid functional. As presented in Table 1, the bandgaps of MoSe_2_ and HfS_2_ monolayers were 2.02 and 1.99 eV, respectively, which are consistent with the previous calculations [54] and mean that the two layers can both utilize the visible light irradiation. Moreover, the valence band maximum (VBM) and conduction band minimum (CBM) of the HfS_2_ monolayer were both lower than those of MoSe_2_ single layer, which indicates that MoSe_2_/HfS_2_ heterobilayer can form a type II heterostructure. As we all know, all of the direct Z-scheme systems have a typical type II band alignment structure [26]. Therefore, the MoSe_2_/HfS_2_ heterojunction can form a direct Z-scheme photocatalyst.

**Figure 2:**
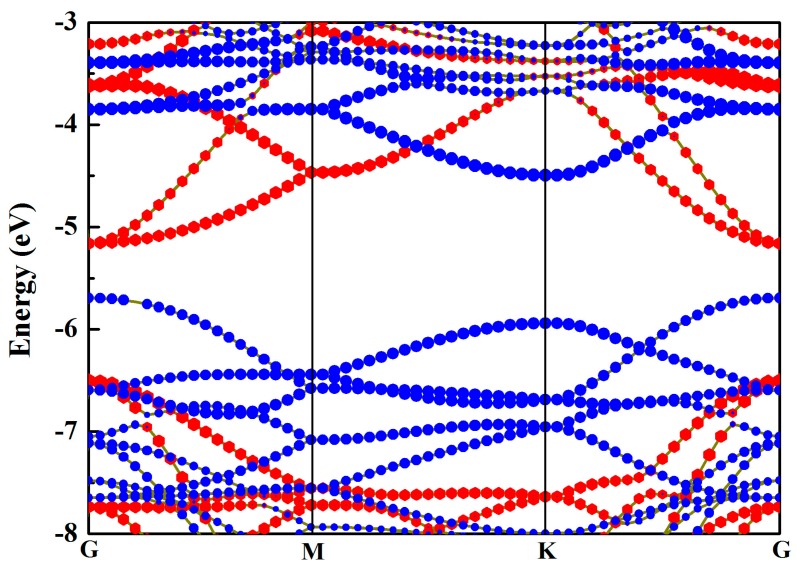
Projected band structure of the MoSe_2_/HfS_2_ heterostructure. The red hexagons and blue balls represent the energy bands of the HfS_2_ and MoSe_2_ layers, respectively.

Meanwhile, the VBM of the HfS_2_ single layer was able to stride over the standard oxidation potential for O_2_/H_2_O, whereas the CBM was 0.5 eV lower than the standard reduction potential for H^+^/H_2_. So, the HfS_2_ monolayer can only be used for OER. At the same time, the VBM of the MoSe_2_ monolayer was 0.04 eV higher than the standard oxidation potential, while the CBM could straddle the standard reduction potential, indicating that the MoSe_2_ single layer can only be applied for HER. Thus, by combining the MoSe_2_ and HfS_2_ single layers, the MoSe_2_/HfS_2_ nanocomposite can be used for photocatalytic overall water splitting. As depicted in Figure 2, the MoSe_2_/HfS_2_ heterostructure is a semiconductor with a direct bandgap of 0.53 eV, which is less than those of the individual MoSe_2_ (2.02 eV) and HfS_2_ (1.99 eV) single layers, indicating that this heterojunction can make the best of visible light and even enlarge the applied range of light to infrared light. As shown in the picture of the projected band structure, the VBM of this heterostructure is mainly composed of the MoSe_2_ layer, while the CBM is primarily made up of the HfS_2_ layer, which is in support of the separation of the photoinduced carriers.

**Figure 3:**
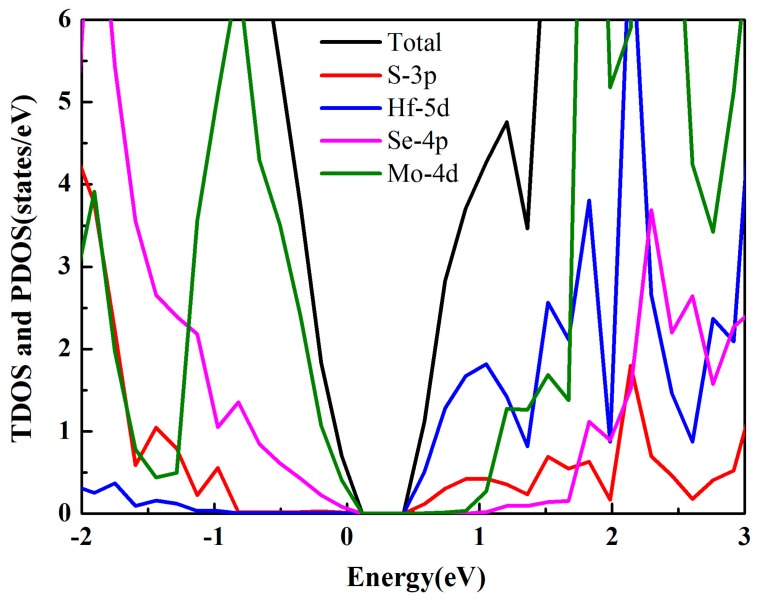
Total and partial density of states of the MoSe_2_/HfS_2_ nanocomposite.

In order to systematically study the photocatalytic ability of the MoSe_2_/HfS_2_ heterostructure, we employed a density of states (DOS) analysis. TDOS and PDOS represent the total and partial DOS, respectively. As displayed in Figure 3, the VBM of this heterojunction chiefly consisted of the Mo 4d and Se 4p states, which were derived from the MoSe_2_ layer. However, the CBM was primarily made up of the Hf 5d and S 3p orbitals, which were rooted in the HfS_2_ slab. Thus, the VBM and CBM of this heterojunction were separated into different layers, which is consistent with the previous analysis about the band structure.

**Figure 4:**
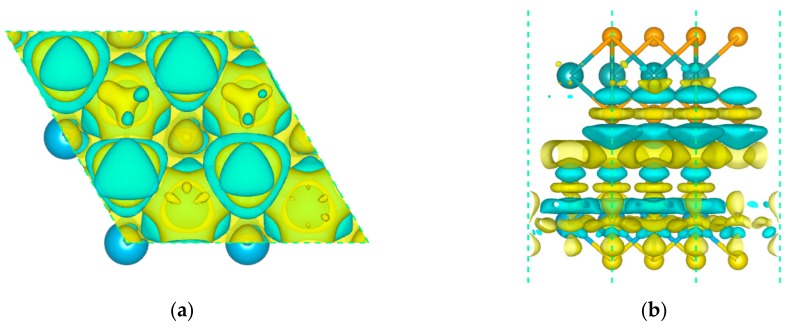
Electron density difference in the MoSe_2_/HfS_2_ nanocomposite with an isovalue of 0.0001 e/Å^3^. Yellow and cyan areas represent accumulation and depletion, respectively. Charge density differences for the MoSe_2_/HfS_2_ heterojunction (top view (**a**) and side view(**b**)).

The effective separation of the photo-generated electron–hole pairs is an essential factor for improving the photocatalytic activity. Thus, we applied the charge density difference and Bader charge analysis to investigate the carrier migrating processes. The charge density differences of this heterostructure are defined as this: △ρ = ρ_MoSe2/HfS2_ − ρ_MoSe2_ − ρ_HfS2_, where ρ_MoSe2/HfS2_, ρ_MoSe2_, and ρ_HfS2_ indicate the charge density of the MoSe_2_/HfS_2_ nanocomposite, freestanding MoSe_2_, and HfS_2_ nanosheets, respectively. The small bandgap (0.53 eV) in this heterostructure was favorable to enhancing the interlayer carrier transfer. As depicted in Figure 4, there is obvious charge accumulation and depletion near the interface. The MoSe_2_ layer was apt to lose charge, while the HfS_2_ slab tended to gain charge. Hence, the charge transfer between two layers brought about an internal electric field pointing from the MoSe_2_ layer to the HfS_2_ slab, which is in accordance with the previous report that the electric field generally points from the hydrogen evolution catalyst to the oxygen evolution catalyst [25]. In order to evaluate the quantity of charge transfer, we employed the Bader charge analysis. The S and Se atoms were liable to acquire electrons, while the Hf and Mo atoms were apt to lose electrons. As a whole, the migrated electrons from the MoSe_2_ layer to the HfS_2_ slab were 0.052 e, which is in keeping with the charge density differences analysis.

**Figure 5:**
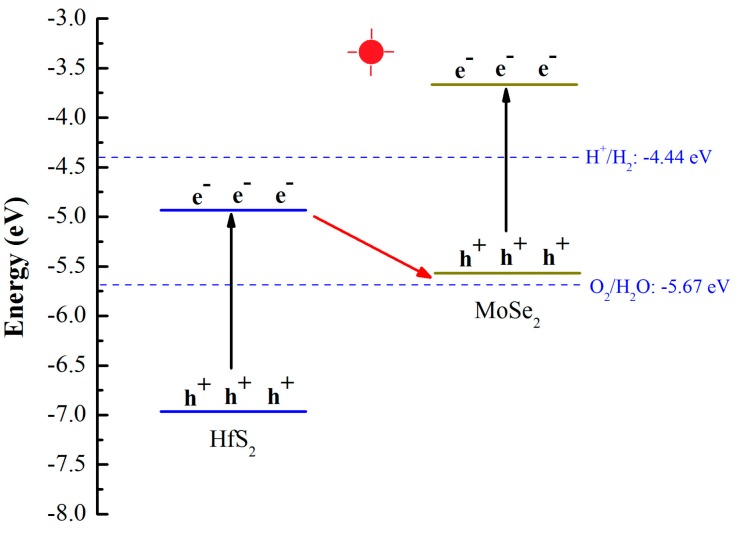
The schematic illustration of the Z-scheme photocatalytic mechanism for the MoSe_2_/HfS_2_ nanocomposite.

After the light irradiation, the electrons on the VB of MoSe_2_ and HfS_2_ slabs were excited to the CB of these layers. The internal electric field of the MoSe_2_/HfS_2_ nanocomposite was in favor of the electron migration from the CB of HfS_2_ to the VB of MoSe_2_. Meanwhile, the electric field hindered the electron transfer from the CB of MoSe_2_ to the CB of HfS_2_ and the hole transfer from the VB of HfS_2_ to the VB of MoSe_2_. Moreover, because the CBM of HfS_2_ and VBM of MoSe_2_ were close to each other (0.69 eV)—as displayed in Table 1—the photogenerated electrons in the CBM of HfS_2_ layer could easily recombine with the holes in the VBM of the MoSe_2_ layer. Finally, the photoinduced electrons in the CBM of MoSe_2_ slab and the holes in the VBM of HfS_2_ slab could both be preserved, which enabled the MoSe_2_/HfS_2_ nanocomposite to possess high redox ability. The schematic illustration of the Z-scheme photocatalytic mechanism for the MoSe_2_/HfS_2_ nanocomposite is illustrated in Figure 5. Therefore, in comparison with the MoSe_2_ and HfS_2_ monolayers, the MoSe_2_/HfS_2_ heterostructure can improve the separation efficiency of the photogenerated carriers and enhance the redox ability.

**Figure 6:**
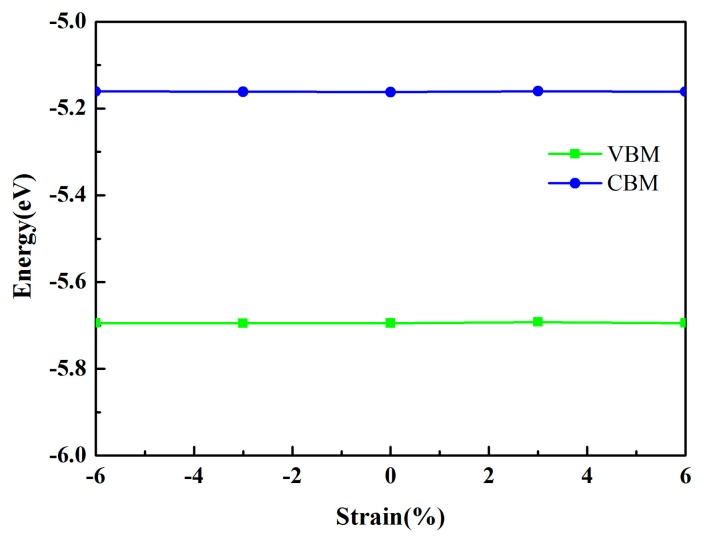
The band edge positions of the MoSe_2_/HfS_2_ nanocomposite as a function of in-plain strains.

In addition, stress is inevitable in industrial production and may also originate from the mismatch of the lattices between different materials. Thus, we investigated the band edge positions of the MoSe_2_/HfS_2_ nanocomposite as a function of in-plain strains. By applying the strains in the range from −6% to 6%, the calculated band edge positions of the MoSe_2_/HfS_2_ heterostructure remained almost unchanged, as displayed in Figure 6. Thus, the band structures of MoSe_2_/HfS_2_ heterostructure are stable, which is different from the typical type II heterojunctions [11,15]. Therefore, the stable electronic property is in favor of future industrial applications.

### 3.3. Optical Properties

**Figure 7:**
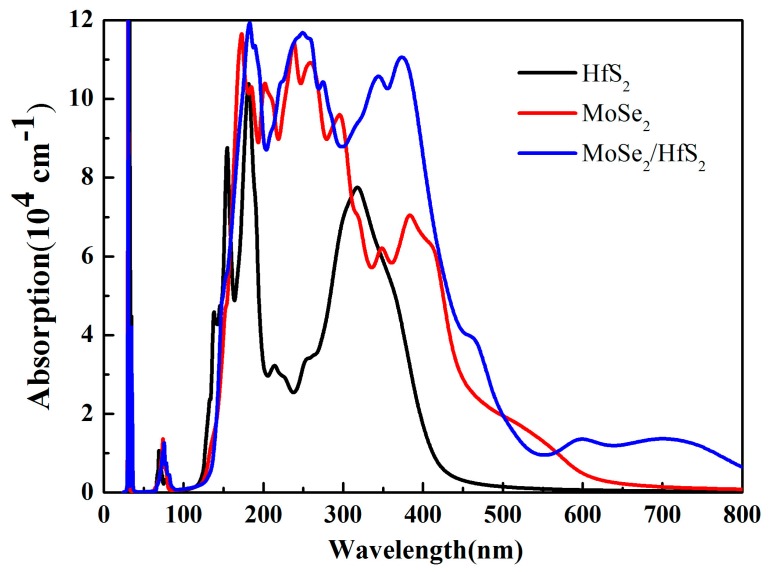
Optical absorption spectra of the related 2D materials and nanocomposite.

Optical absorption spectra are able to directly characterize the catalytic performance of photocatalysts. In this work, we employed the VASPKIT software to analyze the optical absorption properties. The optical absorption coefficient *I(ω)* can be acquired from the dynamical dielectric response function *ε(ω)*, which can be expressed by this equation: $I(\omega )=\sqrt{2}\omega {\left[\sqrt{\varepsilon_{1}{\left(\omega \right)}^{2}+\varepsilon_{2}{\left(\omega \right)}^{2}}-\varepsilon_{1}(\omega )\right]}^{\frac{1}{2}}$. Compared with those of the HfS_2_ and MoSe_2_ monolayers, the optical absorption intensity of the MoSe_2_/HfS_2_ heterostructure is strengthened, especially in the visible and infrared region, as shown in Figure 7. Because the HfS_2_ single layer is an indirect semiconductor, the enhancement of the optical absorption mainly stems from the MoSe_2_ monolayer, which possesses a direct bandgap. Moreover, the evident red shift in this nanocomposite is mainly derived from the transition from the S 3p state to the Se 4p state. Furthermore, there are obvious absorption peaks even at 400 and 600 nm. Therefore, the MoSe_2_/HfS_2_ can take full advantage of the visible light.

## 4. Conclusions

To summarize, we have designed a MoSe_2_/HfS_2_ bilayer heterostructure and investigated its electronic and optical properties according to the direct Z-scheme mechanism by employing the hybrid density functional theory. The computed results reveal that the HfS_2_ monolayer and MoSe_2_ single layer can only be used for OER and HER, respectively. By combining the MoSe_2_ and HfS_2_ monolayers, the MoSe_2_/HfS_2_ nanocomposite, which is a direct Z-scheme photocatalyst, can be used for photocatalytic overall water splitting. As depicted in the projected band structure, the VBM of this heterostructure is mainly composed of the MoSe_2_ layer, while the CBM is primarily made up of the HfS_2_ layer. By applying the charge density difference and Bader charge analyses, the charge transfer between two layers brought about a built-in electric field pointing from the MoSe_2_ layer to the HfS_2_ slab, which is in support of the separation of the photoinduced carriers. Moreover, the photogenerated electrons in the CBM of the MoSe_2_ slab and the holes in the VBM of the HfS_2_ slab can both be preserved, which enables the MoSe_2_/HfS_2_ nanocomposite to possess high redox ability. When strains are applied to the MoSe_2_/HfS_2_ heterostructure, the band structures of this heterostructure are stable, which is in favor of future industrial applications. Compared with those of HfS_2_ and MoSe_2_ monolayers, the optical absorption intensity of the MoSe_2_/HfS_2_ heterostructure is distinctly strengthened in the visible and infrared region. Therefore, the MoSe_2_/HfS_2_ heterojunction is a potential direct Z-scheme photocatalyst for photocatalytic overall water splitting. This work may provide an effective route for developments in clean and renewable energy.

## Figures and Tables

**Table 1:** Bandgaps and band edge positions of the related nanosheets.

Structure	E_g_ (eV)	E_VBM_ (eV)	E_CBM_ (eV)	Bandgap Type
MoSe_2_	2.02	−5.63	−3.61	Direct
HfS_2_	1.99	−6.93	−4.94	Indirect
MoSe_2_/HfS_2_	0.53	−5.69	−5.16	Direct
MoSe_2_/HfS_2_ with −6% strain	0.53	−5.69	−5.16	Direct
MoSe_2_/HfS_2_ with −3% strain	0.54	−5.70	−5.16	Direct
MoSe_2_/HfS_2_ with 3% strain	0.53	−5.69	−5.16	Direct
MoSe_2_/HfS_2_ with 6% strain	0.53	−5.69	−5.16	Direct
